# Growth arrest lines and intra-epiphyseal silhouettes: a case series

**DOI:** 10.1186/1756-0500-7-27

**Published:** 2014-01-10

**Authors:** Ian W Kennedy, Greg J Irwin, James S Huntley

**Affiliations:** 1 The Western Infirmary, Glasgow G11 6NT, UK; 2 Radiology Department, Royal Hospital for Sick Children, Glasgow, Yorkhill G3 8SJ, UK; 3 Glasgow University/Orthopaedic Department, Royal Hospital for Sick Children, Glasgow, Yorkhill G3 8SJ, UK; 4 University of Glasgow, School of Medicine, Glasgow G12 8QQ, UK

**Keywords:** Growth arrest lines, Orthopaedics, Physis

## Abstract

**Background:**

Growth arrest lines can develop within the skeleton after physiological stress or trauma. They are usually evident on radiographs as transverse lines in the metaphyses and have been used in fields from palaeontology to orthopaedics. This report consists of three cases, two of which describe growth arrest lines in an intra-epiphyseal site hitherto rarely documented, and a third demonstrating their clinical application.

**Case presentation:**

Case 1 describes a 9-year-old who suffered a knee hyperflexion injury requiring anterior cruciate ligament and posterior cruciate ligament reattachments. She subsequently developed a marked distal femoral intra-epiphyseal arrest silhouette, as well as metaphyseal arrest lines in the femur, tibia and fibula. Case 2 describes an 8-year-old who sustained a tibial spine fracture and underwent open reduction and internal fixation. Subsequent imaging shows a further example of femoral intra-epiphyseal arrest silhouette as well as tibia and fibula metaphyseal arrest lines. Case 3 describes a 10-year-old who sustained a distal tibia fracture which was managed with open reduction and internal fixation. Subsequently the metaphyseal growth arrest line was parallel to the physis, suggesting no growth arrest (a danger with such a fracture).

**Conclusion:**

This case series describes two examples of rarely described intra-epiphyseal growth arrest silhouettes and demonstrates the usefulness of arrest lines when assessing for growth plate damage.

## Background

Growth arrest lines occur at sites where there is a slowing of longitudinal bone growth, appearing as transverse metaphyseal lines on radiographs [1]. They were first documented by Harris [2] in 1926 and have been utilised as a marker of disease in fields from palaeontology [3] to orthopaedics [4].

Clinically, arrest lines may arise following systemic illnesses such as septicaemia, but also develop at the site of localised trauma, for example following a fracture [1]. This case series describes three patients who were seen following trauma, outlining the aetiology and clinical significance of growth arrest lines.

## Case presentation

### Case 1

**Figure 1:**
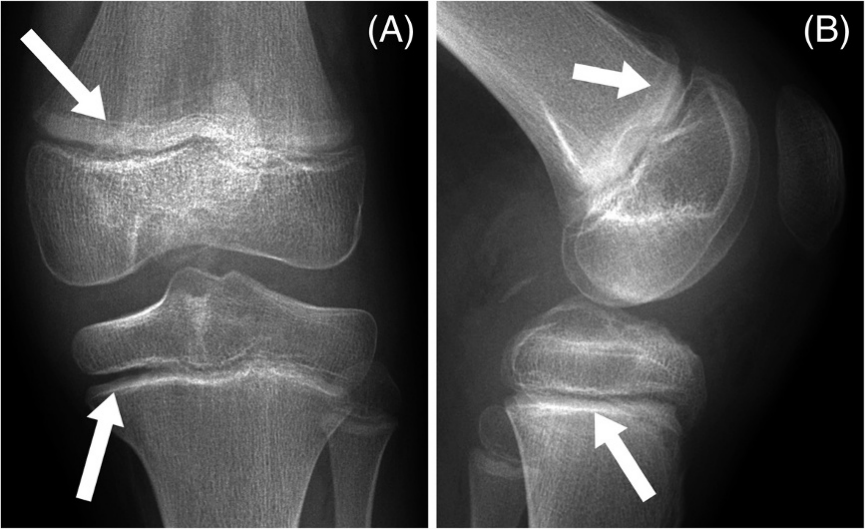
**Anteroposterior (A) and lateral (B) radiographs taken four months after knee injury and operative reattachment of the anterior and posterior cruciate ligaments.** Arrows indicate early metaphyseal arrest lines in the femur and tibia.

**Figure 2:**
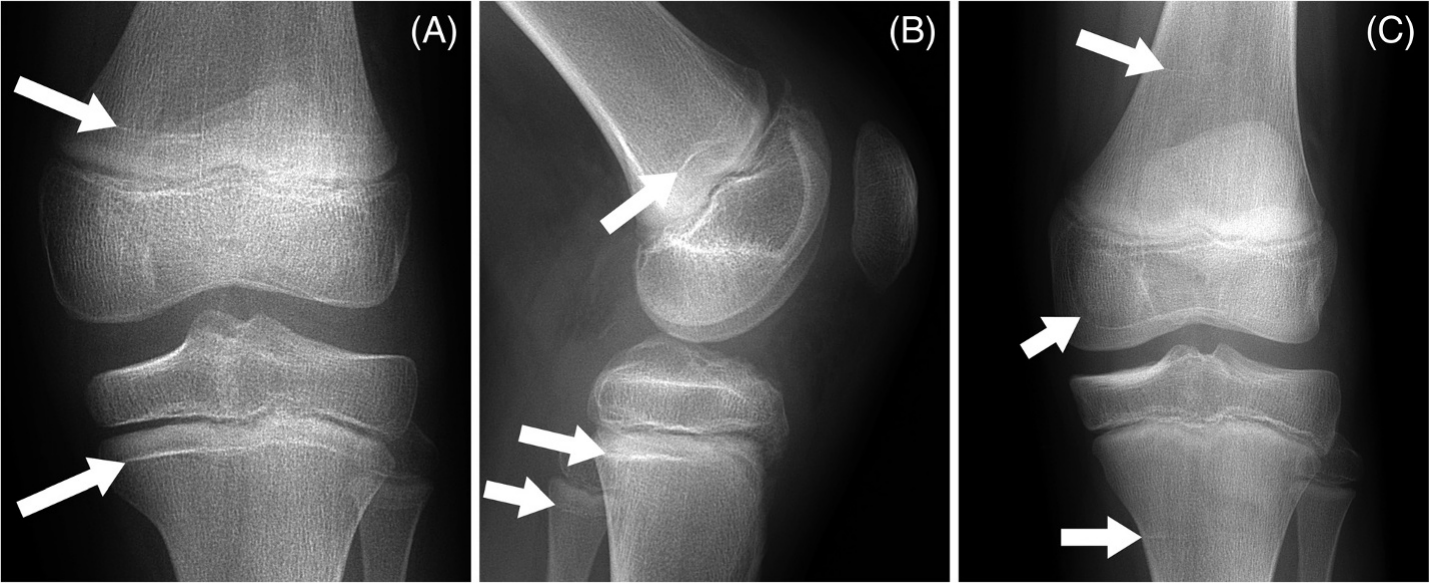
**Anteroposterior (A,C) and lateral (B) radiographs taken six months (A,B) and thirty-one months (C) after knee injury and operation.** Arrows indicate mature metaphyseal arrest lines in the femur, tibia and fibula. The anteroposterior view at thirty-one months after injury **(C)** shows the transverse metaphyseal tibial and femoral arrest lines to be far less marked (arrowed), and to have migrated away in parallel to the physes. The middle arrow indicates the marked intra-epiphyseal femoral growth silhouette.

A 9-year-old girl suffered a left knee hyperflexion injury, resulting in avulsions of her anterior and posterior cruciate ligaments (ACL/PCL). This was managed with ACL/PCL reattachments via an anterior arthrotomy. Post-operative radiographs (Figures 1 and 2) show conventional metaphyseal femoral, fibular and tibial growth arrest lines, but also an intra-epiphyseal distal femoral epiphyseal ‘arrest silhouette’ (Figure 2C).

### Case 2

**Figure 3:**
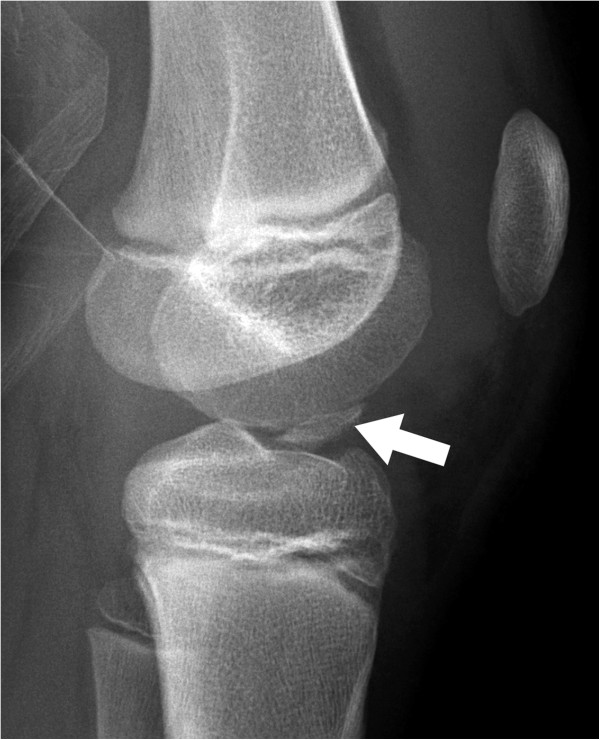
Lateral radiograph displaying the displaced tibial spine (arrow).

**Figure 4:**
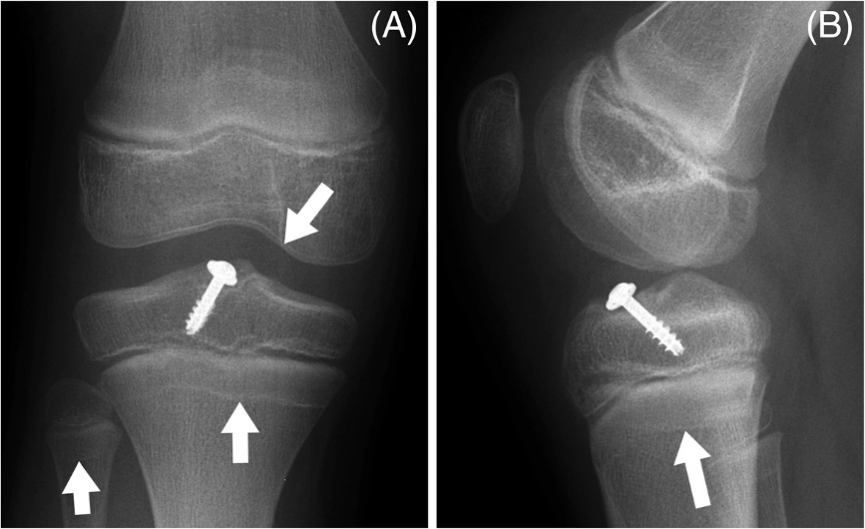
Anteroposterior (A) and lateral (B) radiographs nine months post-fixation showing proximal tibia and fibula transverse metaphyseal arrest lines and a distal femoral intraepiphyseal arrest silhouette.

This 8-year-old girl sustained a right-sided tibial spine fracture (Figure 3). Given its displacement, this required open reduction and internal fixation. Post-operative radiographs (Figure 4) show growth arrest lines in the proximal tibia and fibula but also an intra-epiphyseal distal femoral arrest silhouette.

### Case 3

**Figure 5:**
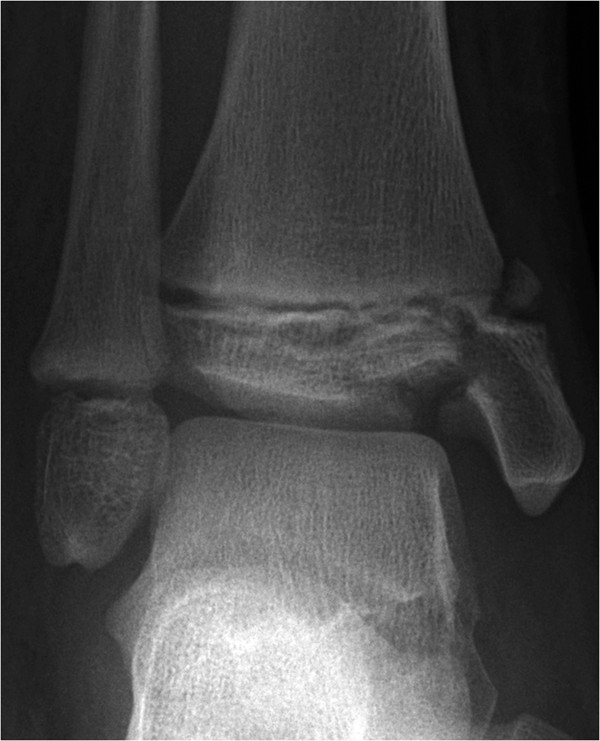
Anteroposterior ankle radiograph showing the comminuted Salter-Harris three distal tibia fracture, with a depressed articular ‘die-punch’ fragment.

**Figure 6:**
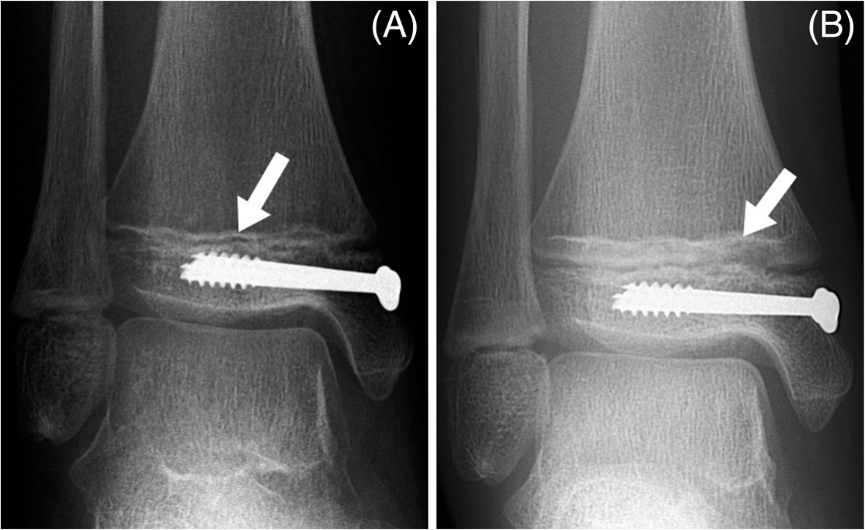
**Anteroposterior ankle radiographs at two (A) and seven (B) months after operative fixation.** The growth arrest line (arrowed) has grown away in parallel from the physis.

A 10-year-old girl suffered a comminuted distal tibial fracture after falling from a height (Figure 5). Given the injury’s close proximity to the physis, despite the anatomical reduction there was a risk of damage to the growth plate with subsequent growth disturbance. Serial radiographs (Figure 6) demonstrate the arrest lines moving away over time in parallel from the physis, demonstrating the absence of growth arrest at this stage.

## Conclusion

When exposed to a period of stress or trauma, a temporary arrest of ossification can ensue. Subsequent removal of the stressor and return to normal growth leaves a growth arrest line in its wake [4]. These can be seen radiographically and are usually most evident in the metaphyses of the distal femur and proximal tibia. These areas are thought to be especially prone to develop such lines because of their constitutively rapid growth. Conversely, areas of slower growth such as in the metacarpals and phalanges, display arrest lines less commonly [1]. Here, we have reported two examples of intra-epiphyseal silhouettes which hitherto have been rarely reported. Arrest lines, in the form of a silhouette, have however been described in the patella following recurrent dislocation [4]. Growth slowdown was believed to result from articular trauma rather than impaired blood supply, with subsequent intervention alleviating damage and producing an arrest line. Intra-epiphyseal arrest lines have been documented rarely such as by Oestreich [5] in relation to certain metabolic bone diseases. Here we have documented two instances of intra-epiphyseal growth arrest silhouettes (Figures 2 and 4), occurring secondary to trauma.

Growth arrest lines were first documented by Harris in 1926 [2]. At this time, they were believed to be the result of calcium deposition and were produced experimentally in animals through starvation. Following this, Park [6] (1964) produced similar results in rats through protein and fat deprivation and suggested that the primary cause of line formation was a dissociation between chondrogenesis and osteogenesis. Recent histological examination has demonstrated, however, that the key anatomical change is a deviation in the trabecular orientation from longitudinal to transverse, and this contrast in orientation can be detected radiographically [1]. As transverse trabeculae are found normally in slower growing bones, this indicates ossification is not abnormal but rather occurring at a reduced rate [1]. Furthermore, growth does not stop completely, and even in severe illness ossification can still be demonstrated at the metaphysis [7]. Although long bones demonstrate longitudinal trabeculae normally as a result of their rapid growth, bones such as the metacarpals, which are less dynamic, contain degrees of longitudinal and transverse trabeculae [1,8], thus making the appearance of arrest lines less obvious on radiographs. In addition, these lines alter with bone remodelling. Those closest to the metaphysis are thicker in comparison to lines which have migrated towards the diaphysis, and are less fragmented in appearance. With time, the lines can disappear completely [9].

Although the precise aetiology of growth arrest lines is not fully understood they typically arise during sustained periods of disease or biological stress, for example starvation, septicaemia [7] and chemotherapy [10]. They have therefore been utilised regularly in paleontological studies to assess the health of ancient civilisations by determining their presence or absence in skeletal remains [11]. Some authors have also demonstrated in such skeletal studies a greater mean number of arrest lines in those with a lower age at death [3]. For those with generalised illnesses or undergoing chemotherapy, arrest lines can signify adequate treatment by highlighting the return of normal growth after removal of the stressor [1]. In addition to systemic conditions, they can arise in areas of localised trauma, as demonstrated in the above three cases, and can be utilised to assess for growth plate damage. Should growth plate injury occur, arrest lines can predict the development of bone deformity three months after the initial trauma [12].

In the post-traumatic situation, the relationship of the Harris line to the articular surface should be gauged as well as that to the growth plate [12]. These two relationships may be different if there is a concurrent change in epiphyseal growth pattern [12]. If a bone bar forms at the site of physeal damage/malreduction, the tether can cause both a reduction of normal length achieved and asymmetric growth that causes a progressive angular deformity. Peterson [12] additionally comments that useful growth information may be derived from the Harris lines in companion bones (e.g. in cases 1 and 2 in our report).

Growth arrest lines have been utilised in a variety of fields to demonstrate illness. In the first two cases the appearances of the arrest lines are both metaphyseal and intra-epiphyseal, the latter a position that has only been documented rarely before. In the final case, the position and orientation of the arrest lines is reassuring concerning the state of the distal physis.

## Consent

Written informed consent was obtained from the patients’ guardians for publication of this case series and the accompanying images. Copies of the written consents are available for review by the Editor-in-Chief of this journal.

## Abbreviations

ACL: Anterior cruciate ligament; PCL: Posterior cruciate ligament.

## Competing interests

The authors declare that they have no competing interest.

## Authors’ contributions

JSH managed all patients and conceived of the idea for the report after a literature review. IWK reviewed the literature and wrote the first draft. JSH and IWK together revised and rewrote the manuscript. GJI contributed to the revised manuscript and obtained the images. All authors’ read and approved the final manuscript.
